# Research progress on *Haemophilus parasuis* vaccines

**DOI:** 10.3389/fvets.2025.1492144

**Published:** 2025-02-11

**Authors:** Yu Duan, Yue Hao, Huapeng Feng, Jianhong Shu, Yulong He

**Affiliations:** College of Life Sciences and Medicine, Zhejiang Sci-Tech University, Hangzhou, China

**Keywords:** *Haemophilus parasuis*, Glässer’s disease, vaccines, pathogenesis, *Glaesserella parasuis*

## Abstract

*Haemophilus parasuis* (HPS) is the causative agent of porcine Glässer’s disease, which has become prevalent in China in recent years. It is characterized by fibrinous polyserositis, arthritis, and meningitis, but often shows mixed infection with other upper respiratory tract pathogens, causing heavy economic losses to the pig industry. Vaccination is an important means to prevent and control HPS infection, and the currently available vaccines are mainly the inactivated type or subunit vaccines containing immunogenic HPS proteins. This study reviews recent advances in HPS vaccines, analyzes the relative effectiveness of the components of subunit vaccines and discusses the advantages and disadvantages of each vaccine type. The goal is to provide insights for the development of more effective vaccines against *Haemophilus parasuis* infections in pigs.

## 1 Introduction

*Haemophilus parasuis* (HPS) is an opportunistic Gram-negative bacterium in the *Pasteurella* family that causes upper respiratory tract infections of pigs (1). It is motile and diverse (2, 3) and requires factor V (nicotinamide adenine dinucleotide, NAD) for its growth (4). There are 15 serotypes defined so far with significant differences in pathogenicity: strains of serotypes 1, 5 and 12 are highly virulent, while those of serotypes 6 and 9 are of low pathogenicity (5–7). The strains circulating in China are mainly serotypes 4, 5, 12, 13 and 14 (8). HPS affects both piglets and adult pigs and is generally transmitted via the respiratory system. This bacterium is commonly found in the upper respiratory tract of healthy pigs, but under certain conditions, such as stress, overcrowding, or poor ventilation, it can cause a systemic infection known as Glässer’s disease, with high morbidity and mortality (9). The main manifestations of the disease are fibrinous polyserositis, arthritis, and meningitis (3, 10). The disease is widespread in the swine industry in China and around the world, causing huge economic losses to hog farmers.

HPS is a typical opportunistic pathogen which can cause infection in piglets when their immune system is compromised by infection with other pathogens or environmental stress. Usually, the maternal antibodies and innate immunity are sufficient to prevent severe HPS infection, but the pathogenic factors of HPS are not well understood. Common antibiotics are the usual first line of treatment for Glässer’s disease, but antibiotic resistance has been reported in clinical HPS isolates (11, 12), and prevention by vaccination is preferred. Tests have been done on inactivated vaccines, attenuated vaccines, subunit vaccines, genetically engineered vaccines, and DNA vaccines, and they have all been reported to combat HPS infection to some degree. The major problem with HPS vaccines is low cross-protection against the multiple heterologous serotypes that can have significant differences in virulence. The number of approved and commercially available vaccines are also limited and there have been cases of vaccination failure (13). Immunization of sows with HPS vaccine could affect the nasal microbiota of offspring piglets during the first 15 days of life, by reducing the relative abundance of *Haemophilus parasuis* and modifying the composition of the microbiota to promote species with anti-pathogenic activity (14). No information is available about the effects of the various types of HPS vaccines on the nasal microbiota of piglets. In this article, we review the current literature on advances in *Haemophilus parasuis* vaccines with an assessment of the pros and cons of each vaccine type to provide insights for the development of novel HPS vaccines that are more effective and offer broader coverage of multiple serotypes.

## 2 Pathogenesis of *Haemophilus parasuis*

The pathogenic mechanism of HPS is relatively complex with many virulence factors involving colonization, invasion, and evasion of the host immune defense system. Upon colonizing the lower respiratory tract, HPS effectively evades the host’s immunoglobulin A (IgA)-mediated mucosal defenses by producing IgA proteases (15). In the early stages of infection in pigs, some virulent strains can block phagocytosis by alveolar macrophages and evade antibody-dependent complement-mediated killing (16), causing systemic infection and widespread seroresistance. Some progress has been made in the role of bacterial surface components, toxin proteins and virulence-related transcriptional regulators of HPS, such as lipo-oligosaccharides (LOS), capsular compounds, outer membrane proteins, and virulence-related transcriptional regulatory molecules. LOS have been shown to play an important role in HPS adsorption and invasion of host cells, in addition to inducing inflammatory factors (17, 18). Eberle et al. constructed an HPS capsular mutant strain that lacked CapD, a key gene for the synthesis of capsular polysaccharides, and confirmed that the mutant strain elicited only a weak immune response (19). Zhang et al. found that an outer membrane protein (OMP P2) deletion mutant of the HPS SC096 strain exhibited increased sensitivity to serum due to activation of the classical complement pathway and increased serum IgG content (20, 21), and that OMP P2 was associated with adhesion of porcine alveolar macrophage cell lines, thereby reducing the adhesion of HPS to epithelial and endothelial cell lines and the capacity for *in vitro* invasion (22). Tang et al. revealed that the extracellular serine protease EspP2 promoted adhesion of HPS to the host through the Rap1 signaling pathway (23).

Melniow et al. used gene chip technology to determine transcriptional profiles in response to simulated conditions of the physiological environment of HPS-infected hosts *in vitro*, such as acidity, temperature, pressure and iron limitation (24). They found 75 genes that were involved in regulating HPS protein expression, and most of these genes were involved in synthesis of iron and glucose metabolite transporters, metabolic enzymes, and DNA metabolism-related proteins. Because of the limited availability of free iron ions in the host, HPS takes up iron directly through hemoglobin, transferrin and lactoferrin (25). Álvarez-Estrada mimicked iron restriction in a host naturally infected with serotype 5 HPS and found that the expression of six iron restriction genes (*tbpA*, *tbpB*, *hxuA*, *hxuB*, *hxuC*, and *fhuA*) was upregulated (26). These included porcine transferrin binding proteins, hemophores, and the transporter and receptor of the heme/hemopexin-binding protein (hxu) operon, a receptor for siderophores (27). However, in Melniow’s study, the fhuA gene was not upregulated during infection. This discrepancy might be due to upregulation of the *fhuA* gene that requires two or more factors acting together.

**Figure 1:**
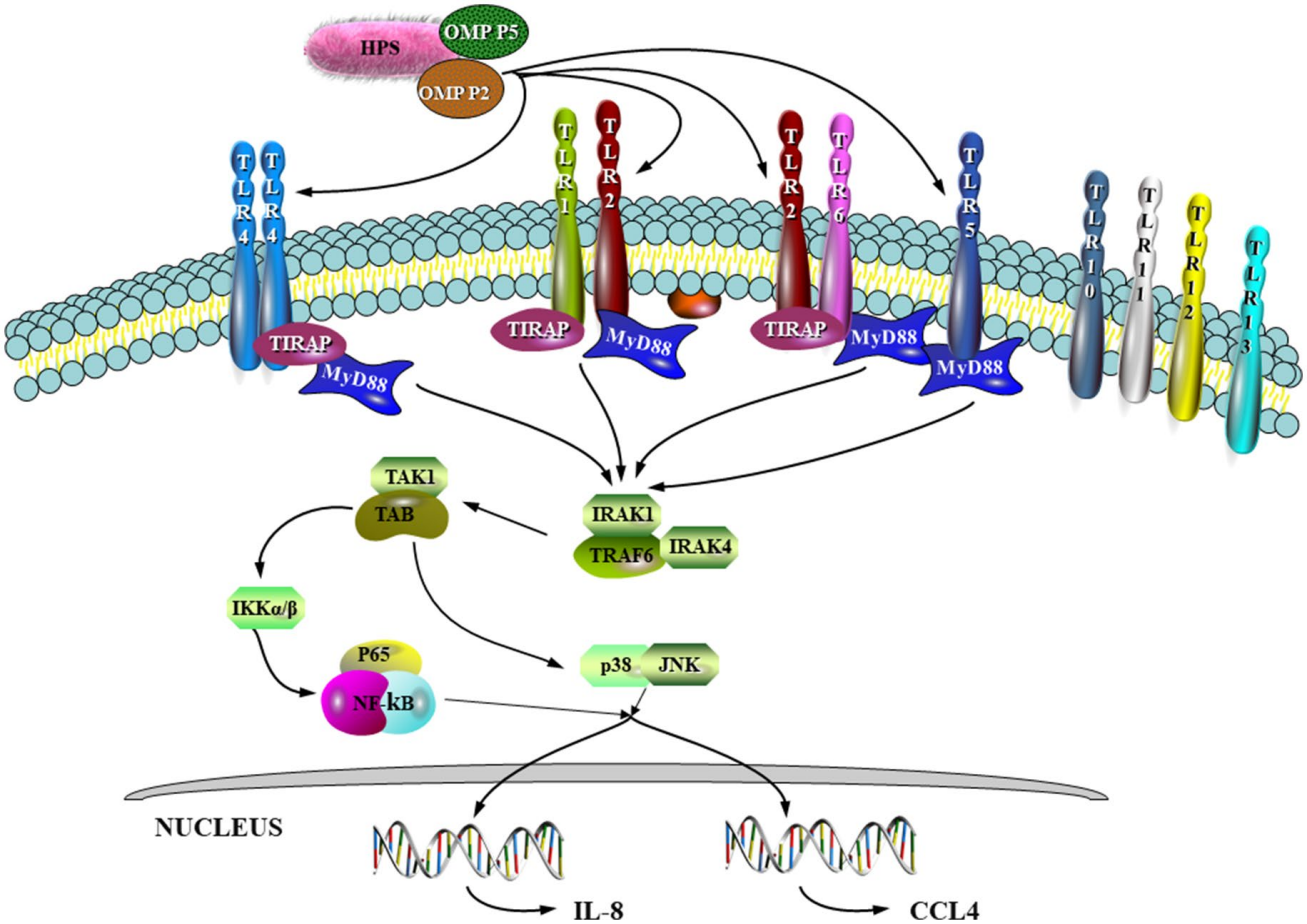
OMP P2 induces IL-8 and CCL4 expression through the NF-κB and MAPK pathways (100), respectively.

HPS infection stimulates host cells to produce signaling molecules through a set of virulence factors, which bind to cognate receptors to activate downstream signaling pathways and promote host production of multiple proinflammatory factors. Earlier studies indicated that the OMP P2 protein of HPS played a key role in pathogenesis by increasing expression of *IL-1α*, *IL-1β*, *IL-6*, and *IL-8* mRNAs in porcine alveolar macrophages (PAMs) and inducing cytokine release in host cells (28–32). Huang et al. reported that excessive and persistent production of proinflammatory cytokines was responsible for severe pulmonary injury in the HPS-infected hosts (33). In the study by Zhou (34), OMP P2 was shown to upregulate mRNA expression of the cytokines IL-17 and IL-23 as well as the chemokines CCL-4 and CCL-5. IL-17. This can lead to excessive inflammation and significant tissue damage due to binding of IL-23 to IL-1, thus maintaining the expansion of Th17 with subsequent release of IL-6, IL-17, IL-22 and TNF-*α* (35–37). In addition, it was further demonstrated that both the surface-exposed Loop7 and Loop8 structures of OMP P2 could induce the transcription and expression of pro-inflammatory cytokines and chemokines such as IL-1*α*, IL-1β, IL-6, IL-8 and TNF-α in PAM and PK-15 by activating the NF-κB and MAPK signaling pathways (38). The diagram in Figure 1, shows that HPS OMP-P2 binds to Toll-like receptors (TLRs) 1, 2, 4 and 6, recruiting linker molecules, MyD88 and TRIF, and linking key proteins TRIF, IRAK4, IRAK1, TRAF6, TAK1 and TAB1, which then activate the NF-κB pathway, inducing the pro-inflammatory factors, IL-8 and CCL4, and activating the p38 and JNK MAPK pathways.

## 3 Vaccines against *Haemophilus parasuis* infection

At present, inactivated vaccines are the most widely used type for *Haemophilus parasuis* prevention, but there are a few subunit vaccines. Highly virulent strains are employed for inactivated vaccines, and these can be monovalent, bivalent, or trivalent, covering single or multiple serotypes. However, inactivated vaccines only show good efficacy against infections of the same serotype, while their protective effect against infections of different serotypes is poor or ineffective. Thus, developing a vaccine with high cross-protection against multiple serotypes is a challenge that needs to be met. Table 1 lists the relevant vaccines currently available.

**Table 1:** Commercial inactivated vaccines against *Haemophilus parasuis*.

Number	Product description	Manufacturer	Efficacy
1	Trivalent inactivated vaccine against the type 4 H4L1, type 5 H5L3, and type 12 H12L3 strains.	Tianjin Ruipu Biotechnology Co. Ltd., China	Protection against serotypes 4, 5 and 12
2	Trivalent inactivated vaccine against the type 4 SH, type 5 GD, and type 12 JS strains.	Spirit Jinyu Biological Pharmaceutical Co. Ltd., China	Protection against serotypes 4, 5 and 12
3	Streptococcal disease-*Haemophilus parasuis* dual subunit vaccine	Wuhan Keqian Biological Co., Ltd.; Tianjin Ruipu Biotechnology Co. Ltd., et al., China	Partial protection against HPS serotypes
4	Porcine circovirus type 2-*Haemophilus parasuis* dual subunit vaccine	Wuhan Keqian Biological Co. Ltd., China	Protection against most serotypes
5	Porcine circovirus type 2-*Haemophilus parasuis* dual inactivated vaccine against the SH, type 4 JS, and the type 5 ZJ strains.	Pleco Bioengineering Co. Ltd., China	Protection against serotypes 4 and 5
6	Trivalent inactivated vaccine against *Haemophilus parasuis* type 4 H25, type 5 H45, and type 12 H31 strains.	Guangdong Yongshun Biopharmaceutical Co., Ltd., China Zhaofenghua Biotechnology (Fuzhou) Co. Ltd., China	Protection against serotypes 4, 5 and 12
7	Trivalent inactivated vaccine against *Haemophilus parasuis* type 4 BJ02, type 5 GS04, and type 13 HN02 strains.	Beijing Huaxia Xingyang Biotechnology Co. Ltd. Sinopharm Animal Health Co. Ltd., China	Protection against serotypes 4, 5 and 13
8	*Haemophilus parasuis* tetravalent propolis inactivated vaccine against type 4 SD02, type 5 HN02, type 12 GZ01, and type 13 JX03 strains.	Shandong Huahong Biological Engineering Co. Ltd., China	Protection against serotypes 4, 5, 12 and 13
9	Dual inactivated vaccine for swine streptococcal disease and *Haemophilus parasuis* LT, MD0322, and SH0165 strains.	Wuhan Keqian Biological Co. Ltd., China	Protection against serotypes 4 and 5
10	Bivalent inactivated vaccine against *Haemophilus parasuis* type 4 JS and type 5 ZJ strains.	Pleco Bioengineering Co. Ltd., China; Luoyang Huizhong Biotechnology Co. Ltd., China	Protection against serotypes 4 and 5
11	Bivalent inactivated vaccine against *Haemophilus parasuis* type 1 LC and type 5 LZ strains.	Shandong Binzhou Wohua Bioengineering Co. Ltd., China	Protection against serotypes 1 and 5
12	Inactivated vaccine against *Haemophilus parasuis* MD0322 and SH0165 strains.	Wuhan Keqian Biological Co. Ltd., China and Zhongmu Industrial Co. Ltd., China	Protection against serotypes 4 and 5
13	Trivalent inactivated vaccine against *Haemophilus parasuis* type 4 H4L1, type 5 H5L3, and type 12 H12L3 strains.	Hunan Zhong’an Biopharmaceutical Co. Ltd., China	Protection against serotypes 4, 5, and 12
14	Inactivated vaccine against *Haemophilus parasuis* type 12 Z-1517 strain.	Boehringer Ingelheim Animal Health Co. Ltd., USA	Protection against serotype 12
15	Inactivated vaccine against *Haemophilus parasuis* type 1 SV-1 and type 6 SV-6 strains.	Hibole Biopharmaceuticals Factory, Spain	Protection against serotypes 1 and 6

In addition, there are two multivalent vaccines applying for clinical trials, namely the porcine circovirus type 2, swine streptococcal disease, *Haemophilus parasuis* triple subunit vaccine filed by Wuhan Keqian Biological Co. Ltd., China; and the swine pseudorabies-*Haemophilus parasuis* dual inactivated vaccine against TY-ΔgE, type 4 BJ02, type 5 GS04, and type 13 HN02 strains filed by Beijing Shengtaier Technology Co. Ltd., Beijing Huaxia Xingyang Biotechnology Co. Ltd., and Huaxia Xingyang (Jiangsu) Biotechnology Co., Ltd., all based in China. Although there are many vaccines available on the market, the disease is still widespread. The reason for this could be that most of the existing vaccines are the inactivated type against serotypes 4, 5 and 12, which do not have good protective efficiency against other serotypes. *Haemophilus parasuis* has less impact than other diseases such as *Mycoplasma pneumoniae*, porcine circovirus disease, and African swine fever, and the swine industry gives priority to vaccination against diseases with greater impact in order to save costs and avoid the need for multiple vaccinations. Therefore, vaccination against *Haemophilus parasuis* is not considered a priority. Thus, it is particularly important to design a vaccine that offers good protection efficiency against the most prevalent serotypes, and the simultaneous immunization against *Haemophilus parasuis* and other diseases will provide the missing incentive for vaccination against lower priority diseases.

### 3.1 Inactivated vaccines

Inactivated vaccines are currently the most widely used against *Haemophilus parasuis*, and these are mainly based on the combination of bivalent or multivalent vaccines of highly virulent serotypes such as type 1, 4, 5, 12 and 13, or multiple vaccines together with porcine circovirus disease, swine streptococcal disease and other porcine epidemic diseases. The results showed that high levels of neutralizing antibodies were produced about three weeks after vaccination with inactivated strains (39, 40), and that piglets born from sows after two inoculations had higher levels of maternal antibodies, which could produce early immune protection (7, 41, 42). In 2001, Takahashi (43) developed an inactivated vaccine against serotypes 2 and 5 and evaluated its safety and efficacy in laboratory and field experiments, where it showed high immune protection against infection by serotypes 2 and 5 along with significant reduction of clinical symptoms in response to challenge infection. Martin (44) prepared an inactivated vaccine in Spain against serotypes 2, 4 and 5 that provided 100% protection in piglets and induced higher levels of cytokines *in vivo* than those of the subunit vaccine group containing outer membrane protein and transferrin-binding protein. Inactivated vaccines against HPS serotypes 4 and 5 have been reported to reduce mortality and clinical symptoms in piglets after challenge with strains of serotypes 4, 5, 13 and 14; however, it did not protect piglets from serotype 12 infection (45, 46). Zhao et al. (47) assessed the efficacy of the trivalent inactivated vaccine for HPS serotypes 4, 5 and 12 in piglets, and the results showed that the protection was 100%.

Although inactivated vaccines still dominate the market, they do have some limitations. Inactivated vaccines cannot contain all disease-causing serotypes at the same time, and the cross-protection efficiency is low. According to one study (48), it is possible to provide heterologous protection against different serotypes if differences in antibody specificity and ability to induce antibody fixation of complement are taken into consideration. Cross-protection against different strains can also be achieved by choosing the proper target proteins as components of subunit vaccines.

### 3.2 Genetically engineered subunit vaccines

Compared with traditional vaccines, subunit vaccines only contain part of the antigenic components of pathogenic bacteria or viruses, eliminating the possible side effects caused by irrelevant components and greatly improving the safety of vaccines (49). At present, two subunit vaccines of *Haemophilus parasuis* have been approved for marketing, namely *Streptococcus suis*-*Haemophilus parasuis* Dual Subunit Vaccine jointly developed by Wuhan Keqian Biological Co. Ltd., China and other units, and Porcine Circovirus type 2-*Haemophilus parasuis* Double Subunit Vaccines. The main antigenic HPS component in these two vaccines is the PalA protein, and the latter also contains HPS 06257 protein. These two antigenic proteins are the outer membrane proteins of *Haemophilus parasuis*, and the other outer membrane proteins, such as D15, OmpP2, OppA, HPS-0675 and GAPDH, have been confirmed as potential candidate antigens with good immunogenicity (49). Related studies have shown that HPS 06257 and PalA proteins are well-conserved in different HPS serotypes. These two proteins can induce high-level specific antibodies *in vivo* and provide good immune protection in the piglet challenge models. Importantly, the PalA protein is highly similar to the P6 protein of *Haemophilus parasuis*, and D15 is highly similar to D15 of *Haemophilus parasuis* and OMA87 of *Pasteurella multocida* (50, 51) as revealed by the proteomes of diverse microorganisms (52). The P6 protein is involved in the immune response during *Haemophilus influenzae* infection (53, 54). These characteristics provide strong support for these two proteins as effective target antigens for an HPS subunit vaccine.

In addition to the subunit vaccines that have been successfully approved for marketing, a number of other recombinant proteins associated with HPS immunogenicity are being studied for potential use in vaccines. Recombinant PilA (rPilA) binds to PK-15 cells, porcine tracheal epithelial cells, and the extracellular matrix components, laminin and fibronectin. The rPilA can react with convalescent and ultra-immune serum of Glässer’s disease patients (55). Purified rPilA elicited a robust immune response and produced strong immune protection against HPS serotype 5 challenge in a murine model. Transferrin-binding protein B (TbpB) also showed outstanding performance in a series of animal immunogenicity challenge and evaluation experiments, and was considered the most promising antigen for the formulation of a subunit vaccine with broad-spectrum protection against HPS (56–58). Jia et al. (59) screened the isolates of serotype 13, cloned the lpxC- and gmhA-related genes, and these proteins induced high levels of IgG antibodies and an immune response with secretion of IL-4, IL-10, and IFN-*γ* in mice. They found that immunization with GmhA and LpxC together could stimulate the production of both Th1 and Th2 immune responses, while recombinant LpxC and inactivated bacteria could only produce a Th2 immune response. In terms of protection from HPS challenge in mice, immunization with recombinant LpxC or GmhA individually resulted in 50% protection, while the combination, LpxC + GmhA, provided 60% protection against infection with a lethal dose of HPS. Dai et al. (60) tested the immunoprotective effect of vaccination with the recombinant polyamine transporter, PotD, in mice and showed that this protein could effectively stimulate both humoral and cellular immune responses. PotD immunization enhanced lymphocyte proliferation, and triggered a Th1-type immune response, protecting the mice from a lethal HPS infection and possibly conferring resistance to HPS colonization. Álvaro et al. (61) evaluated the protective effect of a vaccine containing the three recombinant proteins, rOmpP2, rOmpP5 and rOmpD15, against infection by the HPS Nagasaki strain in piglets lacking colostrum. All three recombinant proteins were recognized and induced specific antibodies in pig serum, but they were not sufficient to protect pigs from HPS challenge under experimental conditions.

With the development of next-generation sequencing technology, availability of a host of complete genomes has made it possible to use the strategy of reverse vaccinology for development of subunit vaccines on a large scale. The basic strategy of reverse vaccinology begins with the bioinformatics analysis of genome-wide genetic information of the target pathogens, and the prediction of potential antigens. The main steps include screening and analysis of genomic information (62), identification of open reading frames with unknown functions (63, 64), homology alignment, subcellular localization of unknown functional ORF-encoded proteins, and functional annotation to identify surface proteins and toxin proteins (65). Comparative genomics and pan-genomics (66, 67) can help researchers respond more efficiently to genetic mutations and immune evasion due to differences between pathogen strains. The precursor LolA protein which comprises the HPS outer membrane lipoprotein carrier protein, and the two HPS outer membrane proteins, RlpB and VacJ (68, 69), were identified by bioinformatics methods as candidates for immunoprotective studies of *Haemophilus parasuis.* The results showed that mice could produce high levels of IgG antibodies in response to immunization with recombinant LolA, resulting in 50% protection against challenge with the virulent strain HPS01, serotype 13. LolA was found to induce Th1 and Th2 immune responses in mice as shown by the level of cytokines of IL-4, IL-10 and interferon-*γ*. The recombinant proteins RlpB and VacJ induced strong antibody responses and high IFN-γ levels in the immune sera of inoculated animals, but they did not afford sufficient protection of the pigs in the challenge test. Li et al. (49) identified three outer membrane proteins TolC, LppC and HAPS_0926 by bioinformatics methods. Mice immunized with these three outer membrane proteins produced humoral and host cell-mediated responses with significantly increased antigen-specific IgG level and lymphoproliferative responses. CD4+ and CD8+ T cells as well as three cytokines (IL-2, IL-4, and IFN-*γ*) were significantly increased in immunized mice. Antisera against the candidate antigens were effective in preventing HPS from surviving in a whole-blood survival assay. It is generally perceived that multi-component subunit vaccines induce a more pronounced immune responses than single-component vaccines.

In addition to the above-mentioned candidate antigens for subunit vaccines, the antigenic proteins listed in Table 2 have also been shown to provide partial protection in HPS challenge experiments. It should be noted, however, that in the majority of these trials, the immunized animals were challenged with highly virulent strains, requiring the highest degree of protection.

**Table 2:** Antigenic HPS proteins that provide partial protection.

Antigenic components (ref)	Species	Protective Effect
Outer membrane proteins P2 and P5 (101)	Mice	Partial protection
Trimeric autotransporters (VtaA) (102)	Piglet	Partial protection
Transferrin-binding protein A (TbpA) (45)	Piglet	Partial protection
Secreted proteins (PflA, Gcp, Ndk, HsdS, RnfC, HAPS_0017) (103)	Mice	Partial protection
GAPDH, OmpA and HPS-0675 (104)	Piglet	Partial protection
HbpA (105), OppA, HPS-04307 and AfuA (106)	Piglet	Partial protection
Extracellular serine protease (Esp P2) (107)	Guinea pigs	Partial protection
High-temperature requirement A (HtrA)-like protease (108)	Mice	Partial protection
Glutathione-binding protein A (GbpA) (109)	Mice	Partial protection

Among the candidate antigens, the OmpA family of OMPs function as adhesins and invasins in the respiratory system that bind to airway epithelial cells, which constitute the principle defensive barrier in the lung. Their detection of pathogens like HPS through Toll-like receptors results in the activation of signaling pathways and release of antimicrobial and pro-inflammatory molecules. VtaA was selected as a candidate antigen for its passenger domains with an extensive mosaic structure and serum cross-reactivity among VtaA from different strains. Most of the remaining candidate antigens that have high immunogenicity were identified through bioinformatics screening.

Subunit vaccines are being widely studied, because they have the advantage over live vaccines of avoiding the risk of HPS dissemination and potential side effects from viral components. Candidate proteins play vital roles in nutrient uptake and virulence, so blocking them can repress HPS infection (70), but there are also problems such as poor immunogenicity and cross-protection against heterologous serotypes. Although the subunit vaccines tested so far have shown promising protective effects in mouse and piglet models, there are other potential protective antigens that remain unexplored. Future development of multi-component subunit vaccines should look for common antigens in different serotypes for cross-protection. Extensive research is still needed, and the choice of proper adjuvant (s) to improve the immunogenicity of subunit antigens is equally important.

### 3.3 Bacterial ghost vaccines

Bacterial ‘ghosts’ (BGs) are empty bacterial particles obtained by the evacuation of cytoplasm and nucleic acids by gentle biological or chemical perforation (71). BGs are a new type of inactivated bacterial vaccine, which maintains antigenicity but is safer because of the absence of cytoplasm and nucleic acids (72). BG vaccines can retain a host of antigens and protein epitopes with adjuvant and drug delivery properties (73), and can induce a stronger immune response because of the native conformation of the epitopes on the BG surface. Hu et al. (74) constructed a BG vaccine from the HPS serotype 5 strain using phage bacteriolytic technology which produced stronger antibody responses in immunized piglets, higher levels of IFN-*γ* and IL-4, and more CD4+ T lymphocytes than the inactivated vaccine. However, the researchers did not conduct an in-depth investigation of cross-protection. There are few studies on BG vaccines of *Haemophilus parasuis* due to the difficulty of their production and relatively high cost, although the stronger protective effect could greatly reduce livestock losses to pig farmers from Glässer’s disease.

### 3.4 Live attenuated vaccines

Live attenuated vaccines are primarily based on manipulation of the main virulence factors by live-passaging or genetic deletion, which make them less- or non-pathogenic, but still immunogenic. Compared with inactivated vaccines, stable live attenuated vaccines can induce longer-lasting immune responses in the body and are important vaccine candidates for *Haemophilus parasuis* (75). In 2020, Eberle (19) demonstrated that the HPS HS069 mutant with deletion of the cap gene (polysaccharide biosynthetic protein and glycosyltransferase protein) had enhanced biofilm formation ability compared with the wildtype, could effectively adhere to 3D4/21 cells, and had reduced resistance to macrophage phagocytosis. This study not only confirmed the important role of capsular polysaccharides in HPS infection in piglets but also demonstrated how a genetic deletion could be used to generate an attenuated vaccine. Cytolethal distending toxin (CDT) is an important virulence determinant of many bacterial pathogens that acts by blocking the cell cycle. Zhang et al. (21) constructed an attenuated strain of HPS SC096 by deleting the *CDT* gene and found that the mutant exhibited reduced adherence to and invasion of porcine umbilical vein endothelial cells (PUVEC) and a porcine renal epithelial cell line (PK-15). Deletion of the *rfaE* gene, a core biosynthetic enzyme gene in LOS, an important virulence factor of HPS, reduced adhesion to PUVECs and PK-15 cells by 10-fold and 12-fold, respectively (76). In the study of Lin et al. (77), piglets infected with the wild-type JS0135 strain exhibited more significant tissue damage and pathological changes compared to piglets infected with a ΔCDT mutant. In addition, Lin et al. demonstrated that loss of the CDT gene cluster in JS0135 led to increased susceptibility to phagocytosis by porcine alveolar macrophages (3D4/2) than that of the wild-type strain. The two-component system senses the density modulator QseBC, which plays an important role in the virulence of the *Enterobacteriaceae* and *Pasteurella* families (78). Yan et al. (79) constructed a ΔQseBC mutant of HPS SC1401 and infected porcine alveolar macrophages and mouse alveolar epithelial cells (MLE-12) with this mutant. They found that the ability of the mutant to adhere to and invade PAM and MLE-12 was significantly reduced. In the mouse challenge model, the mortality rate of mice inoculated with HPS SC1401 was 87.5%, while that of mice inoculated with ΔQseBC was only 50%. Pathological and histological examination of spleen and lungs showed that the pathological effects of ΔQseBC were milder than those of the wild-type group, indicating that the deletion mutant weakened the virulence of HPS in mice. In addition, deletion mutants of capD, cheY, hfq, wza, and lgtF genes have all been reported to have reduced pathogenicity.

Compared with inactivated vaccines, live attenuated vaccines have the advantage of reducing the risk that inactivated vaccines are not completely inactivated and still partially infective. They can distinguish between infected and vaccinated animals, and have a relatively long immune protection period. The primary limitation of live attenuated vaccines is that the main virulence factors and associated pathways of HPS are still not fully understood. With continued in-depth research, live attenuated vaccines are expected to become more widely accepted as a highly effective vaccination strategy against *Haemophilus parasuis*.

### 3.5 DNA vaccine

Compared to subunit vaccines and live attenuated vaccines, DNA vaccines are safer, more stable, and easier to prepare. Fu et al. (80) constructed a novel DNA vaccine (pCgap) encoding GAPDH and explored its immunoprotective efficacy in mice. They showed that the DNA vaccine was highly expressed in mammalian cells and could induce significant humoral immunity as well as adaptive Th1 and Th2 responses in mice. In the mouse model the pCgap DNA vaccine provided 83.3 and 50% protection against challenge with HPS MD0322 and SH0165 strains, respectively. Since the pCgap gene is quite conserved in HPS and is widely present in 15 serotypes, the vaccine has the potential to be protective against all serotypes and is expected to be a vaccine candidate with high cross-protection efficacy. But it also has certain limitations: DNA vaccines may not elicit robust immune responses, requiring additional measures to enhance their effectiveness (81, 82).

### 3.6 Other vaccines

With the development of reverse vaccinology and immunoinformatics, different types of vaccines have become more available. In addition to conventional inactivated vaccines and attenuated vaccines, multi-epitope vaccines are also developing rapidly. A methodology called pan-genomics analysis is used to identify the core genome of a pathogen and predict the B cell and T cell epitopes of the outer membrane proteins encoded by the core genome (83). With this approach, the genes encoding a multi-epitope vaccine could be identified, the antigenicity and physicochemical properties of the proteins could be predicted, and the three-dimensional structure, molecular docking, and molecular dynamics could be simulated (84, 85). Pang et al. (86) retrieved the complete genomes of 105 HPS strains and extracted 8 core genes and their protein sequences using the Roary (87) program for pan-genomic analysis. Next, they designed a multi-epitope vaccine with high scores through linker ligation according to the predicted signal peptide (4), subcellular localization (88, 89), T cell epitope prediction (90, 91), the immunogenicity and physicochemical properties of the antigen (92), and its secondary and tertiary structure (93–95). HPS strains of different serotypes or unclassifiable HPS could be combined to produce polyclonal antibodies in mice after immunization, and the serum levels of IgM + IgG and IgG1 + IgG2 in mice were significantly increased after the second and third immunization, and high levels of B cell populations, cytotoxicity, and T helper lymphocytes were observed, indicating that a cell-mediated immune response was activated. Preliminary evidence suggests that the protein is a promising vaccine candidate.

Multi-epitope vaccines are composed of epitopes of several antigens with high immunogenicity, which solves the problem of low immunogenicity caused by a single antigen and is predicted by computer simulation. Multi-epitope vaccines have been widely used in various animal and human vaccine research, and compared with traditional vaccines, the cost is low. In addition to low production cost and high safety, they can also be composed of a variety of immunogenic peptides of different pathogens to generate protective immune responses to these pathogens as well. However, there are certain limitations. Epitope prediction analysis is typically based on linear sequences and does not consider the influence of the spatial conformation of proteins. With the advancing capability for AI-assisted in-depth interdisciplinary approaches of bioinformatics and multi-omics, the application of multi-epitope vaccines for preventing Glässer’s disease has great prospects (96).

## 4 Conclusions and perspectives

In summary, this review provides a general overview of six types of *Haemophilus parasuis* vaccines. Table 3 summarizes the advantages and disadvantages of each type, showing a clearer and more intuitive comparison of their differences.

**Table 3:** Advantages and disadvantages of different types of vaccines.

Number	Vaccine type	Advantages	Disadvantages
1	Inactivated	Mature technology and wide range of applications	Low cross-protection
2	Subunit	Safe and no side effects	Poor immunogenicity
3	Bacterial ghost	Good immunogenicity	Process is difficult and costly
4	Live attenuated	Long-term protection	Virulence factors poorly characterized
5	DNA	High cross-protection effect	Poor immunogenicity
6	Multi-epitope	Low cost and safe	The structures of many antigens remain unknown

*Haemophilus parasuis* is an opportunistic pathogen in the upper respiratory tract of pigs that usually have co-infections with a variety of viruses, bacteria and mycoplasmas, resulting in severe respiratory diseases. Such co-infections render diagnosis and treatment of the diseases more difficult and bring serious economic losses to the swine industry (97). The approach to effectively prevent and control HPS infection relies on accurate diagnosis, biosafety measures and good vaccines (98, 99). Although the research results obtained so far show good protective efficacy of inactivated and subunit vaccines, the problem of low cross-protection still exists because HPS has multiple serotypes. Therefore, by multicomponent vaccines derived from different serotypes to enhance the cross-protection capability of a vaccines and inclusion of effective adjuvants to boost immunogenicity should be major goals in developing vaccines against HPS infection.
